# Simultaneous measurement of energy spectrum and fluence of neutrons using a diamond detector

**DOI:** 10.1038/s41598-022-16235-x

**Published:** 2022-07-14

**Authors:** Jie Liu, Haoyu Jiang, Zengqi Cui, Yiwei Hu, Haofan Bai, Tieshuan Fan, Jinxiang Chen, Yuan Gao, Xiangjun Yang, Guohui Zhang

**Affiliations:** State Key Laboratory of Nuclear Physics and Technology, Institute of Heavy Ion Physics, School of Physics, Peking University, Beijing, 100871 China

**Keywords:** Experimental nuclear physics, Techniques and instrumentation

## Abstract

Due to the excellent radiation hardness and high–temperature endurance, diamond detectors are suitable for intense neutron measurements and promising for neutron diagnostics of scientific fusion devices. In the present work, simultaneous measurement of energy spectrum and fluence of neutrons using a diamond detector was realized for the first time. The absolute response matrix of the diamond detector was simulated based on detailed analysis of the nuclear reactions and the proper selection of nuclear reaction data. Neutron energy spectra as well as neutron fluences for 5.0, 5.5, 8.5, 9.5 and 10.5 MeV neutrons from d–d reaction were measured using the diamond detector based on the absolute response matrix. The measured neutron energy spectra and neutron fluences are reasonable compared with those detected using a EJ-309 liquid scintillator and a ^238^U fission chamber, respectively, which verifies the reliability of the present work. Furthermore, the energy spectrum and fluence of a 14.2 MeV d–t neutron source were also measured using the diamond detector. The present work demonstrates the ability of simultaneous measurement of energy spectrum and fluence as well as for both d–d and d–t neutrons using a diamond detector, which is of great significance for neutron diagnostics of scientific fusion devices.

## Introduction

The diagnostics of intense neutron field of fusion plasmas has become an interesting and challenging subject^1–3^ as several scientific fusion devices, such as ITER^4,5^, EAST^6^, etc., came into work. Gas filled detectors, scintillators and semiconductor detectors are commonly used for neutron detection. Compared to gas filled detectors and scintillators, semiconductor detectors have the advantages of compact in size, fast charge collection and high energy resolution in neutron detection. However, semiconductor detectors based on silicon and germanium materials cannot meet the requirements for intense neutron detection at high temperature environment in fusion devices^3,7^. A promising alternative is the chemical vapor deposition (CVD) diamond detectors developed in recent decades. Compared with other detectors, diamond detectors are outstanding in radiation hardness^7–9^ and high-temperature endurance^10^. These characteristics make the diamond detectors particularly suitable for the detection of intense neutron fields. For diamond detectors, many studies have been performed in more than two decades including the detector development^11^, performance testing^12^, simulations and measurements of the response functions for neutrons^13–17^, etc. Diamond detectors are also used as an active target to measure the cross sections of neutron induced nuclear reactions of carbon isotopes^17–20^. However, to our knowledge, simultaneous measurement of neutron energy spectrum and neutron fluence using a diamond detector has not been realized up to now.

For measurements of fusion neutrons using diamond detectors in previous studies, d–d fusion neutrons (~ 2.5 MeV) were detected based on the ^12^C(*n*, *el*)^12^C reaction and d–t fusion neutrons (~ 14.0 MeV) were detected based on the ^12^C(*n*, *α*_0_)^9^Be reaction^13,14^, separately, which were both realized by analyzing the pulse height spectra. Simultaneous measurement of d–d and d–t fusion neutrons using diamond detectors is of great significance. The determination of systematic and absolute response matrix of the diamond detector is the key approach for such measurements.

Neutrons are detected through the emission of charged particles from neutron induced reactions. Accurate and absolute response matrix with a wide range of neutron energy makes it possible to obtain the energy spectrum and fluence of an unknown neutron field through deconvolution of the measured pulse height spectrum. However, it is rather challenging to obtain reliable response matrix with a wide neutron energy range and small neutron energy bins. On one hand, existing measurements of neutron response matrix for diamond detectors are limited by the energy range and the energy broadening of neutron sources^13–17,21^. On the other hand, the simulation of the response matrix is a delicate and difficult task because of the numerous and complicated nuclear reactions of ^12^C and ^13^C induced by neutrons, especially the multi-body reactions such as the ^12^C(*n*, *n* + 3*α*) reaction.

In this work, the absolute response matrix of a diamond detector with neutron energy region from 1.0 to 20.0 MeV was simulated using a self-developed code with comprehensive inclusion of nuclear reactions on carbon and appropriate selection of nuclear data from various evaluation libraries. The energy spectra and fluences of the neutrons from d–d reaction with energies of 5.0, 5.5, 8.5, 9.5 and 10.5 MeV were measured using a diamond detector, and then they were compared with those measured with a EJ-309 liquid scintillator and a ^238^U fission chamber. In addition to the d–d neutron source, a d–t neutron source was also measured with the diamond detector to demonstrate its capabilities for fusion neutron diagnostics.

## Simulation of the response matrix

**Figure 1:**
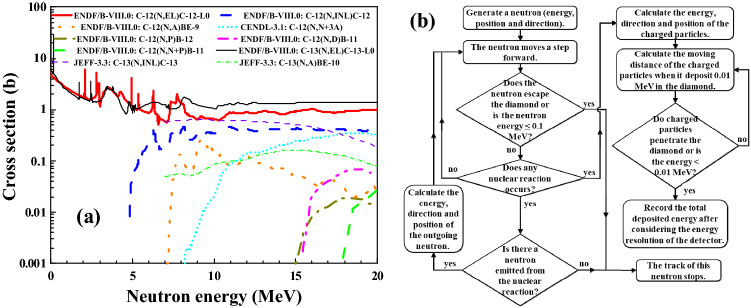
(**a**) The excitation functions of neutron induced reactions on ^12^C and ^13^C in the energy region from 1 to 20 MeV. (**b**) The flow diagram of the simulation of the response matrix of the diamond detector.

Natural diamond is composed of carbon with ^12^C 98.93% in abundance and ^13^C 1.07% in abundance. The inclusion of the nuclear reactions induced by neutrons is critical in the simulations of the absolute response matrix. In the present work, ten reactions (seven for ^12^C and three for ^13^C) are considered in the neutron energy region from 1.0 to 20.0 MeV, as shown in Table 1. The *Q* value refers to the energy released from the nuclear reaction. The nuclear data libraries used in the present simulation are also shown in Table 1. In addition to the cross sections data, the angular differential cross sections are used for two-body reactions, and the double differential cross sections are used for multi-body reactions as shown in Table 1. The excitation functions of the ten reaction channels are shown in Fig. 1a.

**Table 1:** The nuclear reaction channels, corresponding *Q* values and evaluated nuclear data libraries used in the simulation.

Reaction channel	*Q* value (MeV)	Cross section	Angular differential cross section	Double differential cross section
^12^C(*n*, *el*)^12^C	0	ENDF/B − VIII.0 library^22^	ENDF/B − VIII.0 library^22^	Not used
^12^C(*n*, *inl*)^12^C-L1	− 4.439	ENDF/B − VIII.0 library^22^	ENDF/B − VIII.0 library^22^	Not used
^12^C(*n*, *α*_0_)^9^Be	− 5.702	ENDF/B − VIII.0 library^22^	Calculated by Talys − 1.9 Code^25^	Not used
^12^C(*n*, *n* + 3*α*)	− 7.275	CENDL − 3.2 library^23^	Not used	CENDL − 3.2 library^23^
^12^C(*n*, *p*)^12^B	− 12.587	ENDF/B − VIII.0 library^22^	Calculated by Talys − 1.9 Code^25^	Not used
^12^C(*n*, *d*)^11^B	− 13.732	ENDF/B − VIII.0 library^22^	Calculated by Talys − 1.9 Code^25^	Not used
^12^C(*n*, *n* + *p*)^11^B	− 15.956	ENDF/B − VIII.0 library^22^	Not used	CENDL − 3.2 library^23^
^13^C(*n*, *el*)^13^C	0	ENDF/B − VIII.0 library^22^	ENDF/B − VIII.0 library^22^	Not used
^13^C(*n*, *inl*)^13^C-L1	− 3.089	JEFF-3.3 library^24^	ENDF/B − VIII.0 library^22^	Not used
^13^C(*n*, *α*)^10^Be	− 3.836	JEFF-3.3 library^24^	Calculated by Talys − 1.9 Code^25^	Not used

*el* elastic scattering, *inl* inelastic scattering.

The response matrix is composed of a series of response functions for different neutron energies. The flow diagram of the simulation of the response functions for a specific neutron energy is shown in Fig. 1b, which is realized with MATLAB using the Monte Carlo method. In the simulation, a neutron with specific energy enters the diamond along the direction of *z* axis at coordinate (*x*, *y*, *z* = 0) cm. *x* and *y* are generated randomly from − 2.0 to 2.0 mm. The volume of the diamond is 4.0 × 4.0 × 0.5 mm^3^, the same as that of the diamond detector used in the experiments which will be illustrated in the next section. The neutron moves step by step in the diamond with the step length of 1.0 μm. In each step, whether each nuclear reaction occurs or not is judged by calculating the probability of this reaction within this step based on the cross section.

The energies and directions of the secondary particles are calculated according to the nuclear data and the reaction kinematics. For two body reactions, the emiting angle of the light particle is obtained by sampling according to the angular differential cross section listed in the fourth column of Table 1. Then the energies of the emitting particle and the recoil nucleus can be calculated according to reaction kinematics. For the four-body ^12^C (*n*, *n* + 3*α*) reaction, the energies and the emiting angles of the neutron and two of the three alpha particles are extracted according to the double differential cross section shown in the last column of Table 1. The energy of the third alpha particle is caclulated based on energy conservation, and the emiting angle of this alpha particle is extracted according to the double differential cross section. For the three-body ^12^C(*n*, *n* + *p*) ^11^B reaction, the energies and the emiting angles of the neutron and proton are extracted according to the double differential cross section. The energy of the recoil nucleus ^11^B is caclulated based on energy conservation. The conservation of momentum is not considered in the calculation of the multi-body reactions, which is reasonable because the ranges of the charged particles in the diamond is so short that their emtting angles are insignificant.

**Figure 2:**
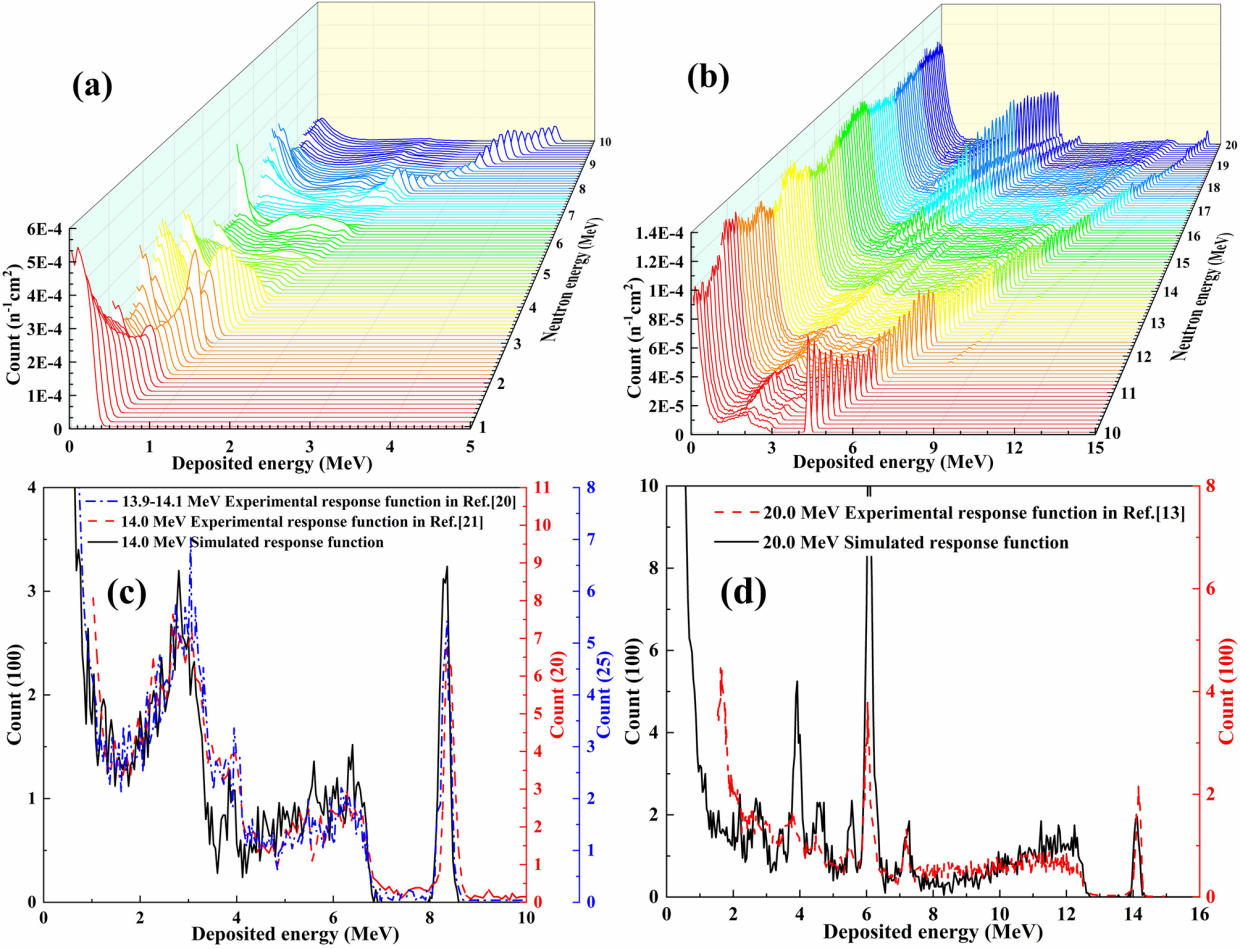
The simulated response matrix of the diamond detector for neutron energies ranging from 1.0 to 10.0 MeV (**a**) and 10.0 to 20.0 MeV (**b**), and the simulated response functions compared with the experimental ones reported previously^13,20,21^ for 14.0 MeV (**c**) and 20.0 MeV (**d**) neutrons.

The charged particles produced in the diamond from nuclear reactions move forward step by step with the energy step of 0.01 MeV until the energy of the charged particle is less than 0.01 MeV or the charged particle moves out of the diamond^26,27^. The total deposition energy of all charged particles from one nuclear reaction is recorded after the correction of energy resolution (4% in the present work). The response function for the specific neutron energy is obtained by finishing the simulations of 4 × 10^6^ incident neutrons. The area of the diamond detector is 4.0 × 4.0 mm^2^, so the neutron fluence through the detector in the simulation is 2.5 × 10^7^ n/cm^2^. The counts of the simulated response functions are then divided by the neutron fluence (2.5 × 10^7^ n/cm^2^) to obtain absolute response function. Finally, the absolute response matrix of the diamond detector is composed by the response functions for neutron energies from 1.0 to 20.0 MeV with an interval of 0.1 MeV, as shown in Fig. 2a,b for neutron energies from 1.0 to 10.0 MeV and 10.0 to 20.0 MeV, respectively. The variations in shape with respect to neutron energy are decided by the *Q* values and the magnitudes of the cross sections for different reactions listed in Table 1. Based on the absolute response matrix, both the energy spectrum and the fluence for an unknown neutron source can be obtained through the deconvolution of the measured pulse height spectrum.

Present response functions are compared with experimental ones reported previously^13,20,21^ for 14.0 and 20.0 MeV neutrons to verify the reliability of the simulation. As shown in Fig. 2c,d, the color of the curves corresponds to that of the ordinates. The overall characteristics of the simulated response functions and the experimental ones are consistent. The local differences in the height of the response functions between simulations and experiments indicate that the accuracy of the nuclear data used in the present simulations needs to be improved.

## Experiments and results

Experiments were performed on d–d and d–t neutron sources separately.

The experiments on the d–d neutron sources were based on the Van de Graaff accelerator at Peking University (PKU) and the HI-13 tandem accelerator at China Institute of Atomic Energy (CIAE). Two neutron energies of 5.0 and 5.5 MeV were measured at PKU and three neutron energies of 8.5, 9.5 and 10.5 MeV were measured at CIAE.

**Figure 3:**
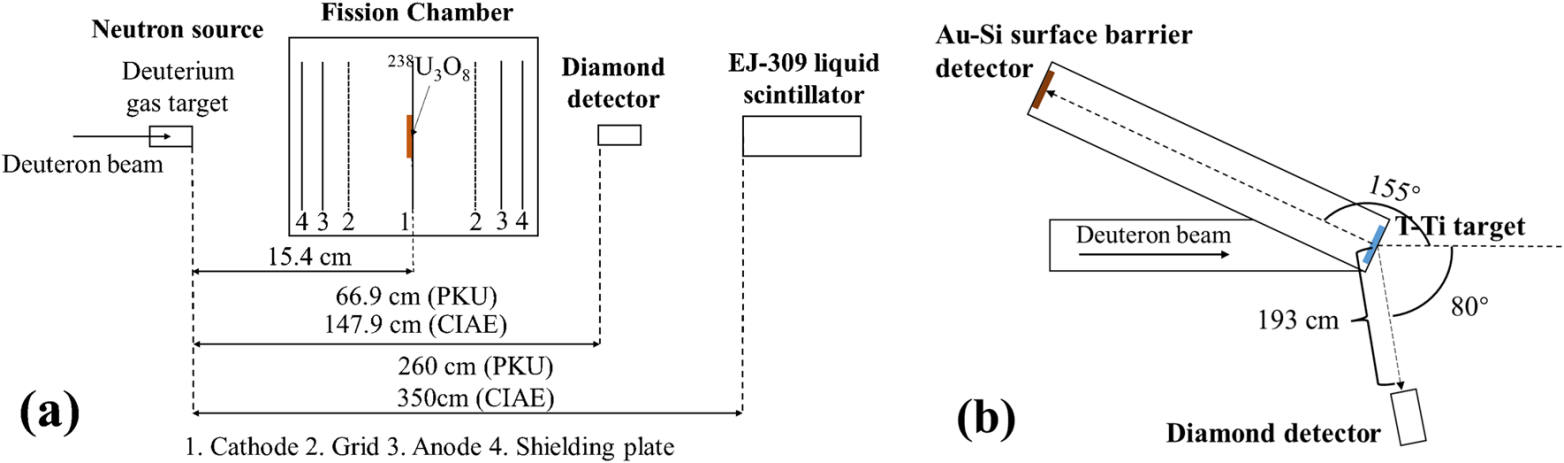
The setup of the experiments based on the d–d neutron source (**a**) and d–t neutron source (**b**).

The setup of the experiments on d–d neutron sources is shown in Fig. 3a including the deuterium gas target^28^, a ^238^U fission chamber, the diamond detector and a EJ-309 liquid scintillator detector. The diamond detector was produced by CIVIDEC corporation. A CIVIDEC fast charge amplifier C6 was used as the pre-amplification^29^. A compound alpha source^28^ was used for energy calibration. The output charge signals were recorded using a commercial CAEN DT5730 digitizer (10 bit, 500 MHz). In addition to the diamond detector, the EJ-309 liquid scintillator and the ^238^U fission chamber were also used to measure the neutron energy spectra and the neutron fluences, respectively, for comparison. Liquid scintillators are commonly used for neutron energy spectrum measurement using the unfolding method^26^, and the ^238^U(*n, f*) reaction as the international standard is generally used for fast neutron fluence determination^30^. The details of the EJ-309 liquid scintillator and the ^238^U fission chamber are presented in Refs.^26^ and^28^, respectively. Combining the relative neutron energy spectra measured from the EJ-309 liquid scintillator and the fluence of the main-energy neutron determined from the ^238^U fission chamber, the neutron energy spectra with absolute fluence can be obtained.

The experiment on the d–t neutron source was based on the Cockcroft-Walton generator at CIAE, the setup of this experiment is shown in Fig. 3b. The deuteron beam with the energy of 300 keV was incident on a solid tritium-titanium (T-Ti) target of 1.0 mg/cm^2^ in thickness. The diamond detector was placed 193 cm from the T-Ti target and 80 degrees with respect to the deuterium beam direction. The energy of main neutrons was ~ 14.2 MeV and the fluence was routinely measured with the associated alpha particle method.

**Figure 4:**
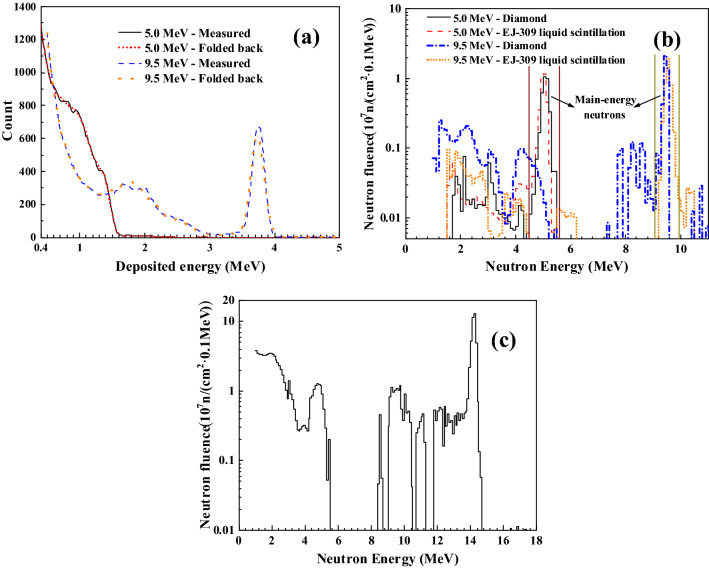
(**a**) The measured pulse height spectra and the folded back pulse height spectra in unfolding with the neutron energies of 5.0 MeV and 9.5 MeV for the d–d neutron sources. (**b**) The neutron energy spectra with absolute fluence measured using the diamond detector compared with those measured using the EJ-309 liquid scintillator together with the ^238^U fission chamber for 5.0 MeV and 9.5 MeV neutrons from the d–d neutron sources. (**c**) The neutron energy spectrum with absolute fluence measured with the diamond detector for the d–t neutron source.

The measured pulse height spectra of the diamond detector are shown in Fig. 4a as two examples for neutron energies of 5.0 and 9.5 MeV. From the measured pulse height spectra and using the GRAVEL iterative unfolding method^31^, the neutron energy spectra and fluences were obtained. And then the folded back pulse height spectra can be calculated through the convolutions of the obtained neutron spectra with the response matrix. Two examples of the folded back spectra are also shown in Fig. 4a. The consistency between the folded back and the measured pulse height spectra indicates the reliability of the response matrix and the unfolding method.

It should be stressed that the energy spectra and fluence of neutrons are obtained simultaneously after unfolding, and two examples of which are shown in Fig. 4b for 5.0 and 9.5 MeV mono-energetic neutrons. The neutron energy spectra and fluence for 5.0 and 9.5 MeV neutrons measured with the EJ-309 liquid scintillator and the ^238^U fission chamber are also plotted in Fig. 4b for comparison. One can see from Fig. 4b that the main-energy neutron peaks determined with the diamond detector and the EJ-309 liquid scintillator together with the ^238^U fission chamber are in good agreement. In addition to the main-energy neutrons, low-energy neutrons measured with the two kinds of detectors are also in agreement. For the energy spectrum of 5.0 MeV neutrons, the low-energy neutron components are mainly from the scattering of the neutron hall. For the energy spectrum of 9.5 MeV neutrons, the low-energy components below 6.0 MeV mainly come from the ^2^H(*d*, *np*)^2^H reaction^32^. This is the first simultaneous measurement of neutron energy spectra and fluences based on the diamond detector.

Results of neutron fluences measured with the diamond detector, the ^238^U fission chamber, the EJ-309 scintillator and the associated alpha particle method are compared in Table 2. As shown in Table 2, the main-energy neutron fluences measured with the diamond detector and the ^238^U fission chamber for all the neutron energies are consistent. The proportions of the low-energy neutrons measured by the diamond detector agree well with those measured with the EJ-309 liquid scintillator for neutron energies of 5.0, 5.5 and 8.5 MeV. However, for neutron energies of 9.5 and 10.5 MeV, the proportions of the low-energy neutrons measured with the diamond detector are higher than those measured with the EJ-309 liquid scintillator. This discrepancy is mainly due to the insufficient precision of the nuclear reaction data used in the simulation at higher neutron energies. Therefore, it is necessary to carry out accurate measurements for neutron-induced nuclear reactions on carbon, especially for higher neutron energies.

**Table 2:** The comparation of the main-energy neutron fluences at the diamond position measured using the diamond detector and fission chamber/associated alpha particle method, and the comparation of the proportions of the low-energy neutrons measured with the diamond detector and the EJ-309 liquid scintillator.

	Deuteron energy (MeV)	Main energy of neutrons (MeV)	Main-energy neutron fluence at the diamond position (n/cm^2^)		The proportion of the low-energy neutrons	
			Diamond	^238^U fission chamber/associated alpha particle	Diamond (%)	EJ-309 (%)
d–d neutron source	2.46	5.0	3.21 × 10^7^	(3.17 ± 0.11) × 10^7^	14.6	16.8
	2.87	5.5	2.99 × 10^7^	(2.92 ± 0.11) × 10^7^	12.6	17.2
	6.00	8.5	3.09 × 10^7^	(2.90 ± 0.11) × 10^7^	25.5	23.5
	6.98	9.5	4.06 × 10^7^	(4.15 ± 0.16) × 10^7^	40.8	29.2
	7.98	10.5	2.02 × 10^7^	(2.05 ± 0.08) × 10^7^	51.2	38.5
d–t neutron source	0.3	14.2	3.75 × 10^8^	(3.62 ± 0.18) × 10^8^	–	–

For the measurement of the d–t neutrons, the energy spectrum with absolute fluence measured using the diamond detector is shown in Fig. 4c. One can see from Fig. 4c that the ~ 14.2 MeV main-energy neutrons as well as low-energy neutrons are measured simultaneously in the neutron energy spectrum. The fluence of the ~ 14.2 MeV neutrons measured with the diamond detector is consistent with that measured with the associated alpha particle method as shown in Table 2. The precision of the spectrum for low-energy neutrons is insufficient, which can be improved by using more reliable nuclear data in simulating the response matrix in the future.

The uncertainty of neutron fluences and neutron energy spectra measured with the diamond detector is not given, because the discrepancy of different evaluated nuclear data for the reactions in Table 1 is too large to analyze the uncertainty.

The above results show that the simultaneous measurement of the neutron energy spectrum and neutron fluence has been realized and the simultaneous measurement of the d–d and d–t neutrons can be realized using a diamond detector.

## Conclusions

Through the comprehensive consideration of the nuclear reactions, detailed analysis of the multi-body nuclear reactions and the appropriate selection of the nuclear reaction data, systematic and absolute response matrix of the diamond detector for neutron energies ranging from 1.0 to 20.0 MeV was obtained based on the Monte Carlo method. Using the GRAVEL method and the obtained response matrix, neutron energy spectra and neutron fluences were simultaneously unfolded from the measured pulse height spectra of the diamond detector for d–d and d–t neutrons. For d–d neutrons, the energy spectra measured with the diamond detector are consistent with those measured with a EJ-309 liquid scintillator, meanwhile, the main-energy neutron fluences measured with the diamond detector are consistent with those measured with a ^238^U fission chamber. These results show that the first simultaneous measurement of the energy spectrum and fluence of neutrons using a diamond detector has been realized. For the d–t neutrons, the energy spectrum with absolute fluence has been measured using the diamond detector, and the fluence of ~ 14.2 MeV neutrons measured with the diamond detector is consistent with that measured with the associated alpha particle method. Our work demonstrates the ability of diamond detectors to simultaneously measure the d–d and d–t neutrons from fusion plasma. These results are of great significance for the neutron diagnostics of scientific fusion devices. The accuracy of the energy spectra and fluences measured with diamond detectors depends on the precision of the nuclear reaction data used in the simulation of the response matrix. Therefore, further measurements and evaluations of related nuclear reaction data on carbon are expected.

## Data Availability

The datasets used and analyzed during the current study are available from the corresponding author on reasonable request.
